# Emodiversity evaluation of remote workers through health monitoring based on intra-day emotion sampling

**DOI:** 10.3389/fpubh.2023.1196539

**Published:** 2023-08-21

**Authors:** Shunsuke Minusa, Chihiro Yoshimura, Hiroyuki Mizuno

**Affiliations:** Center for Exploratory Research, Research & Development Group, Hitachi, Ltd., Tokyo, Japan

**Keywords:** emodiversity, ecological momentary assessment (EMA), experience sampling method (ESM), occupational health, mental health, wearable sensing, well-being, affective computing

## Abstract

**Introduction:**

In recent years, the widespread shift from on-site to remote work has led to a decline in employees’ mental health. Consequently, this transition to remote work poses several challenges for both employees and employers. To address these challenges, there is an urgent need for techniques to detect declining mental health in employees’ daily lives. Emotion-based health assessment, which examines emotional diversity (emodiversity) experienced in daily life, is a possible solution. However, the feasibility of emodiversity remains unclear, especially from the perspectives of its applicability to remote workers and countries other than Europe and the United States. This study investigated the association between subjective mental health decline and emotional factors, such as emodiversity, as well as physical conditions, in remote workers in Japan.

**Method:**

To explore this association, we conducted a consecutive 14-day prospective observational experiment on 18 Japanese remote workers. This experiment comprised pre-and post-questionnaire surveys, physiological sensing, daytime emotion self-reports, and subjective health reports at end-of-day. In daytime emotion self-reports, we introduced smartphone-based experience sampling (also known as ecological momentary assessment), which is suitable for collecting context-dependent self-reports precisely in a recall bias-less manner. For 17 eligible participants (mean ± SD, 39.1 ± 9.1 years), we evaluated whether and how the psycho-physical characteristics, including emodiversity, changed on subjective mental health-declined experimental days after analyzing descriptive statistics.

**Results:**

Approximately half of the experimental days (46.3 ± 18.9%) were conducted under remote work conditions. Our analysis showed that physical and emotional indices significantly decreased on mental health-declined days. Especially on high anxiety and depressive days, we found that emodiversity indicators significantly decreased (global emodiversity on anxiety conditions, 0.409 ± 0.173 vs. 0.366 ± 0.143, *p* = 0.041), and positive emotional experiences were significantly suppressed (61.5 ± 7.7 vs. 55.5 ± 6.4, *p* < 0.001).

**Discussion:**

Our results indicated that the concept of emodiversity can be applicable even to Japanese remote workers, whose cultural background differs from that of individuals in Europe and the United States. Emodiversity showed significant associations with emotion dysregulation-related mental health deterioration, suggesting the potential of emodiversity as useful indicators in managing such mental health deterioration among remote workers.

## 1. Introduction

Remote work, also known as telework, has become one of the popular work styles for white-collar workers (1, 2). Especially in recent years, owing to the COVID-19 pandemic, many organizations have been forced to adopt complete or partial remote work arrangements (3, 4). This transition from on-site to remote work has brought various positive and negative effects depending on workers’ conditions (5–9). As a positive aspect, for example, previous studies reported increased work style flexibility, decreased commuting burden, and the reduction of psychological and physical stress responses owing to a decrease in job stressors (4–6). Conversely, remote workers also faced difficulties in communication and collaboration and felt social isolation and loneliness, sometimes leading to mental health deterioration (6–9). In these ways, the transition to remote work has reduced social communication between managers and employees, as well as between employees, creating challenges for employers in promoting the mental health of employees who work remotely.

One potential solution to address employees’ declining mental health is the use of emotion-based health assessments, as proposed in the field of affective computing (10, 11). Decreased mental health during remote work may be caused partly by a change in the quality of social communication (12, 13). In conventional face-to-face communication, managers can realize emotional depression, as a sign of mental health deterioration, through non-verbal information such as tone of voice and facial expression (14). However, communication under remote working brings about a decrease in communicating non-verbal information (e.g., emotion), which causes the difficulties in detecting signs of worsening mental health (15). If emotion can be assessed by some indicators remotely, it may be possible to detect mental health deterioration or its signs at an early stage, which can lead to early intervention and treatment before the deterioration becomes more severe and chronic.

One of the indicators could include assessing the variety of experienced emotions. Previous psychological studies have suggested that the diversity of emotional experiences in daily life, referred to as emodiversity, may be a helpful indicator of both mental and physical health (16–19). The concept of emodiversity is inspired by biodiversity, and it is one of the measures to quantify how rich and complex our daily emotional lives are (16, 19, 20). High emodiversity is also considered to have the ability to differentiate emotional experiences without being dominated by specific emotions, enabling persons to adapt to various stressors (21, 22). For example, the Midlife in the United States (MIDUS) study measured participants’ daily emotions using a diary once a day (19). They reported the associations between high emodiversity of positive emotions and positive health conditions. A high diversity of positive emotions was also associated with lower biological inflammation (18). Thus, emodiversity-based health assessments may have the potential to monitor the mental health of remote workers.

Despite this possibility, the validity of emodiversity remains controversial and inconsistent depending on experimental conditions (19, 23). This inconsistency may be partly caused by a difference in the population and research methods (18, 19). Most studies have focused on healthy adults or patients with specific symptoms in Europe and the United States (16–19, 24), which probably included few remote workers owing to the rapid increase in remote work only after COVID-19. If the validity of emodiversity depends on population type, the feasibility of using emodiversity to monitor the mental health of remote workers remains unclear, especially for those remote workers whose cultural backgrounds are different. In diary-based studies, participants often recall their daily state inaccurately over time (i.e., recall bias) because our memory is easily affected by the context at reporting time and a few essential episodes (25). Especially regarding emotion, the emotional memories tend to be biased owing to recollection (26). In remote work, workers experience blurred boundaries between work and rest time (6), which can be easily influenced by recall bias. If the recall bias also affects emodiversity, more precise research tools may help to clarify the actual condition regarding emodiversity among remote workers.

To bridge this gap in existing literature, this study aimed to investigate the association between mental health decline, emodiversity-related psychological factors, and physical conditions in remote workers. To determine whether emodiversity can serve as a mental health indicator for remote workers in populations beyond Europe and the United States, we conducted a consecutive 14-day pilot study in Japan. The cultural background in Japan differs from those regions, making it important to investigate this aspect. To ensure a quantification of emodiversity, without recall bias, we implemented the smartphone-based experience sampling method, which is well-suited for collecting context-dependent self-reports in real-life settings (refer to the Materials and method section) (25, 27). We then analyzed psycho-physical characteristics, including emodiversity, on health-declined experimental days.

## 2. Materials and methods

### 2.1. Experimental design and procedures

**Figure 1:**
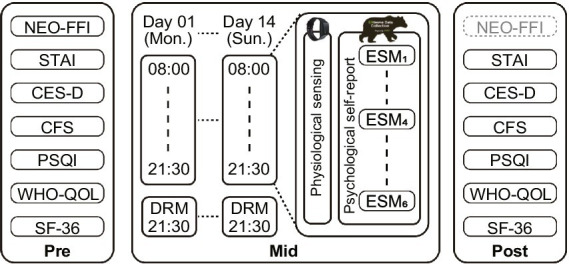
Schematic diagram of the experimental protocol. The experiment was composed of pre-, mid-, and post-terms. During the pre-and post-terms, participants completed several questionnaires reporting their demographics and baseline psychological conditions. During the consecutive 14-day mid-terms experiment, participants reported their physiological data and rated their subjective daily health and emotional conditions.

This study is part of research aiming at developing novel monitoring methods of subjective health deterioration based on physiologically measured data. To this end, we focused on whether psycho-physical factors, especially emotional variations, are associated with subjective health decline in remote workers. The study protocol was approved by the internal review board of Research & Development Group, Hitachi, Ltd., and was conducted in accordance with the Declaration of Helsinki. All participants provided informed consent prior to enrollment in this study. We conducted a consecutive 14-day prospective observational experiment for Japanese remote workers (Figure 1). Our experiment consisted of pre-and post-questionnaire surveys, ambulatory sensing, and smartphone-based surveys to assess subjective psychological states under in-the-wild conditions. The smartphone-based survey involved collecting daytime emotions at various times during the day and daily subjective health at the end of each day.

A total of 18 healthy adult workers were recruited for this study through company bulletin boards. The participants were employees of a Japanese company that offers remote work options. Each participant satisfied the following inclusion criteria: (i) individuals who plan to work remotely in the experimental term; (ii) individuals who have never received treatment for mental illness; (iii) individuals who have never experienced severe skin problems owing to wearing wristband-like devices; and (iv) individuals who can use their smartphones and their messaging apps described later, to collect psychological data. Participants did not receive any financial or non-financial compensation.

Participants in the study completed self-reporting questionnaires before and after the experiment. The following seven questionnaires, as shown in Figure 1, were used to assess various participant traits. The Neuroticism Extraversion Openness Five-Factor Inventory (NEO-FFI) (28) was used to evaluate the big five personality traits. Note that the NEO-FFI was only obtained in the pre-term and was measured using standard scores among the general Japanese population. The State–Trait Anxiety Inventory (STAI) (29) was used to evaluate anxiety. The Center for Epidemiologic Studies Depression Scale (CES-D) (30) was used to evaluate depression. The Chalder Fatigue Scale (CFS) (31) was used to evaluate the degree of fatigue. The Pittsburgh Sleep Quality Index (PSQI) (32) was used to assess sleep quality. The World Health Organization Quality of Life (WHO-QOL) (33) was used to evaluate the quality of life (QoL). The SF-36v2® Health Survey (SF-36^1^) (34, 35) was used to assess general health conditions. All the questionnaires were Japanese versions and were conducted *via* a website using an experience sampling support system (exkuma, Japan Experience Sampling Method Association, Tokyo, Japan). Demographic information such as age and sex was also collected from the participants.

**Figure 2:**
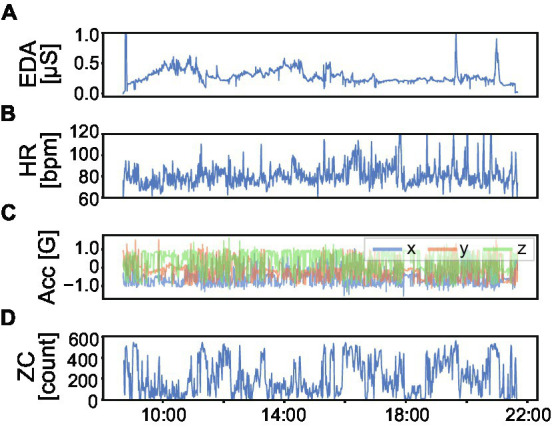
Example of physiological data in one day. **(A)** Electrodermal activity (EDA) **(B)** Average heart rate (HR) **(C)** 3D acceleration (Acc). Blue, red, and green lines represent *x*-, *y*-, and *z*-axis of wrist acceleration, respectively. **(D)** Zero crossing counts (ZC) in 1-min time window calculated from Acc. ZC represents the magnitude of body activity.

During the mid-term period of the experiment, we monitored participants’ physiological data. Participants wore a wristband-type wearable sensor (E4 wristband, Empatica Inc., Boston, MA, USA) on their non-dominant wrist during their waking hours. They were instructed to wear the sensors at all times, except during work-related activities or situations where there was a risk of severe water damage (e.g., bath time). The sensor measured electrodermal activity (EDA) at 4 Hz, photoplethysmography (PPG) at 64 Hz, skin temperature at 4 Hz, and 3D acceleration (Acc) at 32 Hz. In addition, based on the PPG signal, the sensor provided an average heart rate (HR) over 10 s at 1 Hz. It is known that EDA and PPG reflect the activity of the autonomic nervous system, which helps examine changes in emotions (36–38). Therefore, obtaining the above multimodal data allows us to monitor the participants’ physiological conditions (Figure 2).

**Figure 3:**
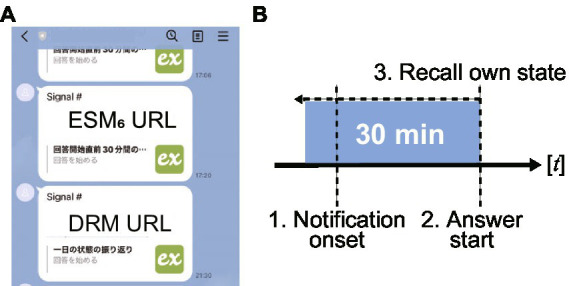
Description of notification-based self-reports. **(A)** Example of user notifications sent to the participant’s smartphone app. Each notification from the experience sampling surveys support system has web URLs to ESM/DRM forms. ESM and DRM notifications are sent 6 times per day and every 21:30 (i.e., 9:30 pm), respectively. **(B)** Timeline for self-reporting. First, notifications are sent to the application. When participants see the notifications and are able to respond, they start self-reporting from the form. In the self-reports, participants recall their own state over the past 30 min and answer the forms.

In addition to physiological data, emotional self-reports were collected from the participants. In previous emodiversity studies, diary-based emotional self-reports were measured every night by recalling emotional states experienced during the day (17). However, in daily life, we experience various emotions depending on the situations we encounter; thus, this recall may not accurately reflect the range of emotions experienced in daily life over time. Due to the inaccuracy of diary-based reports and recall bias, diary-based studies evaluated emodiversity mainly in terms of several-day variations but not intra-day variations. To achieve a more reliable and fine-grained evaluation of emotions based on ecological validity, in the current experiment, emotional self-reports were obtained using the experience sampling method (ESM), also known as ecological momentary assessment (EMA) (25, 27, 39, 40). In ESM, participants respond to self-reports when they receive trigger events on their devices. If we can randomly sample self-reports within a day, we can evaluate participants’ emotional conditions without depending on specific contexts or situations in their daily lives. Considering previous ESM study protocols (12, 39, 41, 42), participants were randomly prompted to respond to six ESM event notifications per day from 8:00 am to 9:30 pm through the experience sampling support system (exkuma, Japan Experience Sampling Method Association, Tokyo, Japan). Notifications were sent to participants’ smartphone apps that are popular in Japan (LINE, LINE Corp. Tokyo, Japan). In addition, reminders were sent for unconfirmed notifications 15 min after the first notification (Figure 3A); for example, the 1^st^ notification was received at 17:06, and the 2^nd^ reminder was received at 17:20 (delayed by a few seconds owing to the system lag). Participants were asked to report their emotional self-reports within 30 min before the start of the response (Figure 3B). Participants were also instructed to respond to the notifications at least three times per day while not disrupting their work. In addition, participants were asked to provide voluntary subjective self-reports when they experienced extreme emotion-evoking events (denoted as an emotional event).

**Figure 4:**
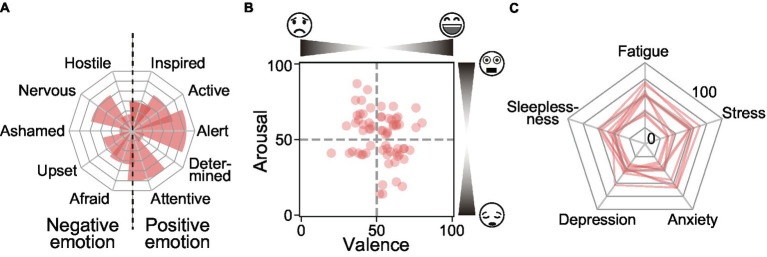
Example of subjective ratings. **(A)** Daily distribution of categorical emotions by I-PANAS-SF in 1 day. The labels on the left side and those on the right side are classified as negative and positive emotion groups, respectively. **(B)** Distribution of some participants’ continuous emotions measured by Affective Slider (44) in all the 14-day terms. Valence and arousal scores are plotted on the *x*/*y* axis, respectively. **(C)** Distribution of some participants’ daily subjective health conditions assessed by VAS in all the 14-day terms. The radial axis represents the severity of fatigue, stress, anxiety, depression, and sleeplessness, respectively.

In emotional rating forms, we measured several types of emotional experiences and their background information. First, participants responded to the International Positive and Negative Affect Schedule Short Form (I-PANAS-SF) (43) to measure the degrees of discrete emotional experiences (Figure 4A). Due to the absence of the Japanese version of the I-PANAS-SF, we used the 6-point scale (ranging from 1 to 6) and instructions used in the Japanese version of the PANAS (45). Next, participants rated the degree of dimensional emotional experiences (i.e., valence and arousal) (46) using the Affective Slider (44), comprising two visual analog scales (VAS) instructed by face scales (Figure 4B). In addition, to capture the contextual information, participants responded to the categories of daily life activities.

To monitor daily mental health conditions, we assessed participants’ subjective mental health scores every night using a day reconstruction method (DRM) measurement. When participants received a notification at 9:30 pm on their smartphone app, they rated their daily health scores across the following five items: fatigue, stress, anxiety, depression, and sleeplessness (Figure 4C). Each score was measured using the VAS ranging from 0 (not at all) to 100 (worst). In addition, participants reported their wake-up time, their in-bed time, and their work styles for the day (i.e., on-site work, remote work, outside work, and holiday).

### 2.2. Data analysis

All 18 participants completed the experiment composed of the pre-, mid-, and post-terms. One participant was excluded due to insufficient data quality. Accordingly, the data of 17 eligible participants (mean ± SD, 39.1 ± 9.1 years) were used in the final analysis (Table 1).

**Table 1:** Descriptive statistics of participants.

Demography	
Participants	17
Male	11
Female	6
Age	39.1 (9.1)
Work styles [%]	
On-site work	17.7 (18.5)
Remote work	46.3 (18.9)
Outside work	1.0 (2.9)
Holiday	35.1 (7.6)

Subjective ratings	ESM	DRM	Emotional event
Total notification	1,392	232	—
Total response	994	221	9
Average response count	58.5 (19.0)	13.0 (1.4)	—
Daily average response rate [%]	71.4 (28.7)	95.2 (21.3)	—

Big five *t*-scores	
Neuroticism	55.3 (9.0)
Extraversion	47.5 (8.1)
Openness	52.8 (10.1)
Agreeableness	51.5 (8.8)
Conscientiousness	45.7 (12.3)

Questionnaire scores	Pre		Post	
	Raw scores	Over thresholds	Raw scores	Over thresholds
CFS score	16.8 (6.0)	—	16.1 (7.9)	—
WHO-QOL score	13.6 (3.6)	8	13.8 (4.2)	6
STAI Trait anxiety	45.1 (10.8)	6	44.6 (9.7)	8
CES-D score	12.0 (8.3)	4	12.9 (8.2)	5
PSQI General score	5.2 (2.3)	7	6.1 (2.5)	8
SF-36 3PCS *t*-score	53.2 (11.5)	—	53.8 (10.9)	—
SF-36 3MCS *t*-score	52.1 (8.0)	—	52.0 (7.3)	—
SF-36 3RCS *t*-score	47.1 (9.1)	—	47.1 (7.8)	—

CFS, Chalder Fatigue Scale; WHO-QOL, World Health Organization Quality of Life 5 form; STAI, State–Trait Anxiety Inventory; CES-D, Center for Epidemiologic Studies Depression Scale; PSQI, Pittsburgh Sleep Quality Index; SF-36, SF-36v2^®^ Health Survey, PCS, Physical Component Score; MCS, Mental Component Score; RCS, Role-social Component Score., Mean (SD) are shown.

We first calculated a daily physical indicator to examine the characteristics of the deterioration of subjective health scores. As a physical indicator, we obtained the daily averaged zero crossing (ZC) count, a measure of body movement known as activity counts in actigraphy (47). We resampled the Acc signal from 32 Hz to 10 Hz and calculated the norm of Acc. After the norm calculation, a 2.0-to 3.0-Hz Butterworth zero-phase filter was applied to the norm signal to obtain a body movement-related signal. We calculated whether the absolute intensity of the filtered signal was greater than 0.01 [G] with a 1-min epoch time, and finally obtained the ZC by taking the daily (i.e., awake time) average. Also, to represent the sleep conditions, we calculated the in-bed time, which is similar to the actual sleeping hours, by using the wake-up time and bedtime measured in DRM.

To represent a variety of emotional experiences throughout the day, we derived emotion-related indicators after validating the representativeness of self-reported data by ESM. While a previous study on emodiversity (17) calculated the indicators of weekly emotional conditions based on daily emotion ratings every night, our study aimed to represent daily emotional conditions by randomly sampling emotion ratings within a day. To ensure the representativeness of the randomly sampled data, it was important to confirm the participants’ response rates and the randomness of the ESM responses within a day. After a preliminary analysis based on previous ESM studies (39, 48), we confirmed that participants were sufficiently compliant with the ESM notifications (for average response count and daily average response rate, see Table 1). Detailed results are mentioned in the Results section below and sufficient randomness of ESM responses within a day is illustrated in Supplementary Figure S1. Based on the preliminary results, we assumed that the obtained ESM data are adequately representative of each day, and accordingly, calculated the emotion-related indicators.

For emotion-related indicators, we obtained the average levels of dimensional emotional experiences and emodiversity of discrete emotional experiences for each day (16, 17, 49). To obtain the average levels of dimensional emotional experiences, we averaged the self-reports of valence and arousal scores for each day (16). To evaluate the diversity of emotional experiences, following the recommendation of a previous study (17), we used the Gini Diversity Index as an emodiversity indicator. The Gini Diversity *G* of participant *i* is defined using the Gini coefficient:

(1) $$GiniDiversity_{i}\equiv G_{i}\equiv 1-\left\{\left(\frac{2\sum_{j=1}^{m}jc_{ij}}{m\sum_{j=1}^{m}c_{ij}}\right)-\frac{m+1}{m}\right\}$$

where c*_ij_* is the count of participant *i*’s emotional experiences within *j* = 1 to *m* discrete emotion categories in ascending order (i.e., *c_ij_* ≤ *c*_*ij* + 1_). Here, we calculated the sum of each score (i.e., 1 to 6) of I-PANAS-SF items (see Figure 4A) within each day, normalized the sum of scores by the number of daily answer counts (i.e., 0 to 6), and obtained *c_ij_* by sorting the answer counts in increasing order. Using *c_ij_*, we finally obtained global *G* (*m* = 10) using all the emotion items, and both negative (*m* = 5) and positive (*m* = 5) *G* by following the negative and positive emotions categories in Figure 4A, respectively.

Using the measured and calculated indicators, we analyzed the associations between subjective health deterioration and psycho-physical factors, especially the variation of remote workers’ emotional experiences in the wild. For each participant, using the participant-wise medians of five mental health-related scores, the 14 experimental days of each participant were grouped into good and bad days for each subjective health score. For example, when we focused on the depression score of participant #A, we first calculated the median depression score of #A for 14 days. Based on the median as a reference, days lower than the median were assigned as good days (healthy days or fewer depression days) in terms of the depression score, and vice versa. We then analyzed whether the subjective health states were associated with the physical factors (i.e., ZC and in-bed time) and the emotion-related psychological factors (i.e., average levels of valence and arousal, and global, negative and positive Gini Diversity *G*). In this analysis, the differences in the participant-wise indicators between the two groups were compared using paired *t*-tests if the data showed normal distribution based on the Shapiro–Wilk test, otherwise, compared using the Wilcoxon signed-rank test.

Data processing and analysis were performed using Python 3.8.13 software, including Scipy 1.6.1. The data are expressed as mean ± standard deviation (SD). Values of *p* < 0.05 were considered to be statistically significant. Statistical significance is denoted as ^*^p < 0.05, ^**^p < 0.01, and ^***^p < 0.001.

## 3. Results

### 3.1. Descriptive statistics

First, we analyzed the descriptive statistics of the participants (Table 1). The participants comprised more male than female participants (male, 11; female, 6). Approximately half of the middle experimental days were conducted under remote work, but the frequency varied widely depending on each participant (Amount of remote work over experimental days, 46.3 ± 18.9%). The participants’ personality traits and SF-36 scores were distributed similarly to the general Japanese population (e.g., Neuroticism of NEO-FFI in standard *t*-score, 55.3 ± 9.0; Physical Component Score (PCS) in *t*-score of SF-36 in pre-term, 53.2 ± 11.5; similar to the Japanese standard, 50.0 ± 10.0). Regarding the other self-reported questionnaires scores, however, several participants indicated higher scores than the cut-off scores of each questionnaire in both the pre-and post-terms (e.g., the number of suprathresholds of the anxiety score cut-off of STAI, six out of 17 participants in pre-term; eight participants in post-term).

Next, we confirmed the condition of the response using the smartphone messaging app. Participants completed the responses to the ESM notifications 58.5 ± 19.0 [times] out of 6 [times] × 14 [days], and those to the DRM notifications 13.0 ± 1.4 [times] out of 1 [times] × 14 [days]. This finding indicates that most participants followed the instruction to respond to ESM notifications at least three times per day. However, regarding emotional events, the response was only 9 [times] across all the experimental periods.

### 3.2. Association between subjective health decline and psycho-physical conditions

**Figure 5:**
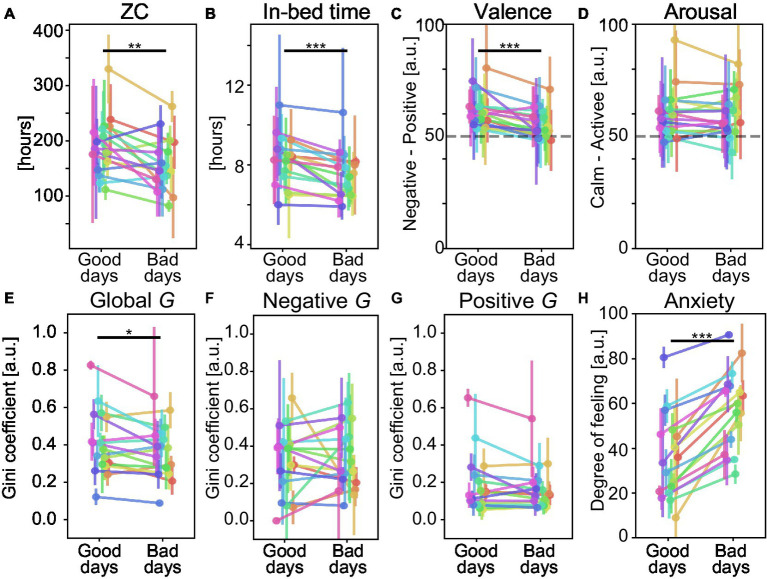
Comparisons between physical and psychological scores on healthy and non-healthy days with respect to subjective daily anxiety. **(A)** Zero crossing (ZC); **(B)** Sleeping hours; Average emotional level of **(C)** valence and **(D)** arousal; Emodiversity based on Gini diversity G. **(E)** Global G, **(F)** Negative G, **(G)** Positive G; **(H)** Reference scores (anxiety) to classify healthy and non-healthy days. Error bars represent the SD of the indices. Each color represents one participant. ^*^p < 0.05, ^**^p < 0.01, and ^***^p < 0.001 by paired *t*-test or Wilcoxon signed-rank test.

**Figure 6:**
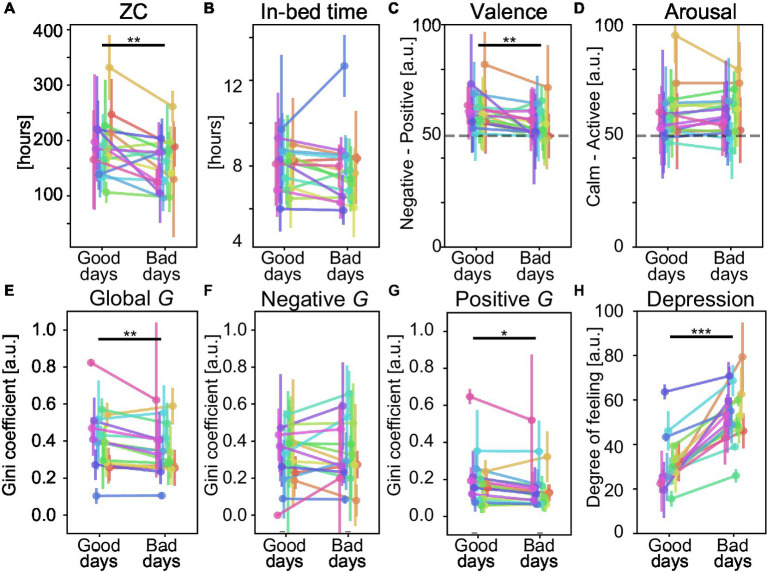
Comparisons between physical and psychological scores on healthy and non-healthy days with respect to subjective daily depression. **(A)** Zero crossing (ZC); **(B)** Sleeping hours; Average emotional level of **(C)** valence and **(D)** arousal; Emodiversity based on Gini diversity *G*. **(E)** Global *G*, **(F)** Negative *G*, **(G)** Positive G; **(H)** Reference scores (depression) to classify healthy and non-healthy days. Error bars represent the SD of the indices. Each color represents one participant. ^*^p < 0.05, ^**^p < 0.01, and ^***^p < 0.001 by paired *t*-test or Wilcoxon signed-rank test.

We analyzed the associations between subjective health decline and psycho-physical factors (Table 2). In the analysis, we divided the experimental days into healthy and non-healthy day groups (i.e., smaller scores and larger scores than the participant-wise median of mental health scores), respectively. For each mental score condition, we compared the between-group difference. The detailed analysis results indicated clear differences in psycho-physical conditions on health declined days, especially regarding daily anxiety (Figure 5) and depression (Figure 6). In addition, the five types of subjective mental health scores often worsened on the same day. For example, when participants reported bad daily anxiety scores, the other four mental health scores also became significantly worse.

**Table 2:** Associations between daily subjective health condition and objective/subjective indicators.

Type		Variable	Grouped by the median of VAS									
			Fatigue		Stress		Anxiety		Depression		Sleeplessness	
			Mean(Diff)	*p* value	Mean(Diff)	*p* value	Mean(Diff)	*p* value	Mean(Diff)	*p* value	Mean(Diff)	*p* value
Daily condition	Reference VAS score	Fatigue	−24.9	—	**−** **15.1**	**<0.001**	**−** **X**	**<0.001**	**−** **8.3**	**0.005**	**−** **6.1**	**0.008**
		Stress	**−** **14.4**	**<0.001**	−24.2	—	**−** **14.7**	**<0.001**	**−** **15.2**	**<0.001**	**−** **11.6**	**<0.001**
		Anxiety	**−** **10.6**	**<0.001**	**−** **16.7**	**<0.001**	−21.8	—	**−** **16.2**	**<0.001**	**−** **9.8**	**0.002**
		Depression	**−** **7.9**	**0.019**	**−** **14.7**	**<0.001**	**−** **12.9**	**<0.001**	−21.8	—	**−** **6.8**	**0.014**
		Sleeplessness	**−** **4.9**	**0.032**	**−** **8.5**	**0.003**	**−** **7.2**	**0.004**	−4.9	0.080	−17.3	—
Physical indicators	Actigraphy	ZC	14.5	0.157	**48.8**	**<0.001**	**35.8**	**0.004**	**33.0**	**0.004**	**23.1**	**0.022**
	Sleep	In-bed time	**0.87**	**0.006**	**0.98**	**<0.001**	**0.79**	**<0.001**	0.26	0.051	**1.00**	**0.026**
Psychological indicators	Average emotion level	Valence	2.9	0.150	**4.6**	**0.008**	**6.0**	**<0.001**	**5.5**	**0.002**	2.9	0.065
		Arousal	−1.8	0.307	−0.3	0.579	0.7	0.517	0.0	0.587	0.3	0.860
	Emodiversity	Global *G*	**0.054**	**0.025**	0.031	0.136	**0.043**	**0.041**	**0.044**	**0.008**	0.026	0.202
		Negative *G*	−0.026	0.441	0.027	0.460	−0.009	0.832	−0.015	0.547	0.036	0.385
		Positive *G*	**0.048**	**0.011**	0.023	0.098	0.024	0.225	**0.030**	**0.009**	0.013	0.632

VAS, Visual Analogue Scale; Mean (diff), Mean (Indicator_Good days_ − Indicator_Bad days_), where experimental days with scores higher than the participant-wise median of each VAS score are labeled as the “Bad days” (non-healthy days) group.; G, Gini Diversity; In-bed time, subjective recording of total sleeping hours [hours]; Grayed variables are the reference indicators to classify whether the health conditions of specific experimental days are good or bad. Bold and underlined variables showed significant differences between good and bad days.

The results showed a significant decrease in physical factors in most of the declining health groups. In terms of the physical activity indicator, ZC decreased significantly except in the fatigue score [e.g., 192.0 ± 51.8 (counts) vs. 156.3 ± 47.7 (counts) in the good days group vs. bad days group, respectively, diff = 35.8 (counts), *p* = 0.004, in the grouping based on the anxiety score; 177.5 ± 42.8 (counts) vs. 163.0 ± 52.1 (counts), respectively, diff = 14.5 (counts), *p* = 0.157, in the grouping based on the fatigue score] (Figure 5A). This indicated that remote workers tended to take fewer physical activities on mental health declined days. The in-bed time, which is roughly considered to be equivalent to the length of sleep, showed a significant decline in non-healthy days except for the grouping based on the depression score (e.g., 8.23 ± 1.27 [hours] vs. 7.44 ± 1.17 [hours] in the good days group vs. bad days group, respectively, diff = 0.79 [hours], *p* < 0.001, in the grouping based on the anxiety score; 7.98 ± 1.03 [hours] vs. 7.72 ± 1.57 [hours], respectively, diff = 0.26 [hours], *p* = 0.051, in the grouping based on the depression score) (Figures 5B, 6B). This also indicated the participants’ shorter length of sleep on mental health declined days.

Regarding emotion-related psychological indicators, the observed associations differed depending on the types of subjective health decline. Throughout the entire experimental period, smaller values of emotions (i.e., strong negative emotions on the Valence scale and strong calm emotions on the Arousal scale) were rarely observed (Figures 5C,D, 6C,D). For decline in the feelings of fatigue, anxiety, and depression, the average levels of valence were significantly suppressed (e.g., 61.5 ± 7.7 vs. 55.5 ± 6.4 in the good days group vs. bad days group, respectively, diff = 6.0, *p* < 0.001, in the grouping based on the anxiety score) (Figure 5C) and emodiversity in global *G* significantly decreased (e.g., 0.409 ± 0.173 vs. 0.366 ± 0.143 in the good days group vs. bad days group, respectively, diff = 0.043, *p* = 0.041, in the grouping based on the anxiety score) (Figure 5E). Moreover, emodiversity of positive emotions significantly decreased during the fatigued and depressed days (e.g., 0.193 ± 0.141 vs. 0.163 ± 0.123 in good days group vs. bad days group, respectively, diff = 0.030, *p* = 0.009, in the grouping based on the depression score) (Figure 6G). By contrast, the average levels of arousal did not show a consistent tendency (e.g., 60.5 ± 11.1 vs. 59.8 ± 9.7 in the good days group vs. bad days group, respectively, diff = 0.7, *p* = 0.517, in the grouping based on the anxiety score) (Figure 5D). Emodiversity of negative emotions also showed no clear associations for mental health deterioration (e.g., 0.319 ± 0.181 vs. 0.328 ± 0.166 in the good days group vs. bad days group, respectively, diff = −0.009, *p* = 0.832, in the grouping based on the anxiety score) (Figure 5F). Regarding the subjective health decline in sleeplessness, no clear emotion-related associations were observed.

## 4. Discussion

The current study examined the associations between subjective mental health decline and psycho-physical factors, focusing primarily on emodiversity among remote workers in real-life settings. Considering the remote work characteristics to easily change surrounding contexts, we introduced an experience sampling-based random sampling of daily emotions for quantifying emodiversity, to relieve the effects of context-dependent recall bias. Our analysis revealed that emodiversity-related indicators were significantly associated with the deterioration of everyday subjective health, especially in anxiety and depression. The findings suggest that emodiversity-based health assessments could serve as valuable indicators to detect deterioration in mental health, even for remote workers. This study also indicates the potential applicability of emodiversity among the Japanese population, whose cultural background differs from that of Europeans and the United States.

We used ambulatory sensing and smartphone-based self-reports to investigate psycho-physical factors in the wild. A previous ESM study (39) focused mainly on students, who reported an average ESM response rate [six (times/day) × five (days)] of 88.9 ± 11.5% under the explicit instruction to require an over-80% completion ratio. Another ESM study for a broad population (42.1 ± 13.0 years; range, 23–63 years) (48) also reported an average response rate of 91.3% in ESM [four (times/day) × five (days)]. Our average response rate appeared to be lower at 58.5 ± 19.0% in ESM, however, this rate still met our minimum requirement of 50% completion on average. Increasing the frequency and interval of notifications could potentially increase the response rates even during remote work. Notably, strong emotional events were less frequently collected through voluntary responses that were not triggered by smartphone notifications. In a previous study that collected strong emotional experiences (42), a total of 954 experiences from 911 days × 11 subjects were collected, suggesting that strong emotions can be collected at a rate of 0.095 [times/days × people]. Compared to this rate, our rate of 0.038 (=9 [times] over 14 [days] × 17 [participants]) [times/days × people] was not extremely low. These findings imply that, in real-life conditions, strong emotional experiences were rare or voluntary responses not prompted by notifications were difficult to respond to. To optimize the collection of intense emotions, it could be beneficial to expand the scale of the experiments and refine our protocols. This may involve implementing measures such as providing incentives to participants.

In the analysis of emodiversity-based factors, our analysis revealed a significant decrease in the average level of valence and intra-day emodiversity in the global Gini Diversity among those experiencing subjective anxiety and depression. We also found a significant decrease in positive Gini Diversity among participants with high depressive days. The decrease in valence was observed in the 50–100 range on the Affective Slider (44), representing the degree of positive emotion. This finding suggests suppression of positive emotions in the non-healthy days group. These characteristics related to positive emotions are in line with the results reported in previous emodiversity research conducted over several days (17, 18), implying the importance of not only inter-day but also intra-day evaluation of the variation in emotional experiences. Studies on anxiety and mood disorders, focusing on the view of emotion regulation, suggest that such disorders may develop or become chronic with diminished positive affect experiences (50, 51) and/or dysregulation of negative affect in daily life (52, 53). Anhedonia, which is the loss of pleasure and motivation, is one of the main symptoms of depression (54, 55). Furthermore, socially anxious persons report fewer subjectively positive events owing to emotion suppression (56). Based on these findings, monitoring emodiversity in real life, especially for positive and negative emotions, can be useful for detecting changes in mental health conditions involving emotion regulation and emotion suppression mechanisms. From this perspective, emodiversity-related characteristics may become the indicators for specific types of mental health decline.

Despite the significant associations between emodiversity indicators and several indicators of decline in mental health, it is important to examine the indicators that did not show significant changes. One such indicator is negative Gini Diversity, which appeared to unexpectedly show large but inconsistent changes across participants (Figures 5F, 6F). In this experiment, the participants did not seem to be in a relatively negative mood from the average valence scores (Figures 5C, 6C), which may simply cause an insufficient evaluation of negative emotions. Although it is difficult to determine the validity of negative emodiversity unless future follow-up studies with sufficient negative emotion sampling are conducted, we may consider this result from another perspective. Previous studies have found that psychological perceptions and responses to moods, including emotions and mental states, vary among individuals based on various factors such as demographics and personality traits (57–59). From this view, it can be interpreted that there is not always a uniform response but rather the existence of subgroups within the associations between emodiversity and poor mental health. If such subgroups are identified, emodiversity indicators may also be helpful as phenotyping tools for understanding and classifying the types of responses to mental health decline (60, 61). In future studies, the identification of such determinants of subgroups should be explored to expand the applicability of emodiversity indicators.

To realize emodiversity monitoring in the form of future possible tools such as digital biomarkers (62), it is necessary to address the challenges of tracking emotional experiences in everyday life. This study employed self-reports to monitor participants’ emotions, which were triggered by random notifications of ESM. However, such frequent self-reporting may become a burden for individuals in their daily lives. Partly owing to this burden, we monitored participants’ emodiversity only for 14 days; however, extended longitudinal monitoring of emodiversity may help in identifying early stages of health deterioration (63–65). To realize such long-term monitoring, alternative low-burden methods of capturing emotional experiences will be required. One possible solution is the use of emotion recognition technology based on physiological sensor data obtained through ambulatory sensing (11, 37, 42). Since emotions are influenced by the autonomic nervous system (36, 38), emotion recognition based on physiological data has shown success in laboratory settings but is currently limited in real-life settings (11, 37, 42). Future improvements in emotion recognition in the wild will enable automatic emotion measurement, reducing the burden of using emodiversity monitoring in real life.

If we can realize emodiversity-related monitoring with an acceptable user burden, various applications can be expected. When we detect early signs of health deterioration through emodiversity-related monitoring, both managers and remote workers themselves can take several countermeasures before workers develop severe mental health symptoms. When managers or employers-associated professionals (e.g., occupational health physicians) detect early signs of health deterioration, they can adjust their subordinates’ workloads, facilitate access to medical care, and grant temporary leave if necessary. Moreover, when the remote workers realize their mental health deterioration, they can receive the emodiversity-based intervention (24, 66) as one of the possible countermeasures. In several types of mental health deterioration, individuals are sometimes dominated by specific emotions without realizing their other non-dominant emotions. Emodiversity is considered to comprise the ability to differentiate emotional experiences (21, 22), which can be trained through mindful sessions (24, 66). Feedback of individuals’ current emodiversity-related status and countermeasures in response to feedback may contribute not only to maintaining better mental health conditions for remote workers but also to improving their well-being.

Although the present study reveals important findings, it has several limitations. One limitation involves the lack of knowledge regarding the association between mental health and physiological conditions. We measured EDA and PPG to assess the activity of the autonomic nervous system, which mainly reflects sympathetic and parasympathetic nervous activities (36, 37). However, owing to the need for specific noise reduction for in-the-wild signals, we have yet to comprehensively conduct a detailed analysis concerning physiological conditions (e.g., heart rate variability indicators) in this study. A previous study reported associations between subjective health and average resting heart rate (67), which implying the importance of analyzing physiological signals. A future detailed analysis of autonomic nervous activities regarding the health decline of remote workers will provide pertinent knowledge regarding the use of emodiversity-related indicators. Another limitation is the existing bias in the participants’ demographics. In this study, the participants were recruited from a specific company that changed its work style from on-site to remote work. In addition, the number of male participants was higher than that of female participants, and several participants exceeded the psychological questionnaire cut-offs (Table 1), which indicates a potential inclination toward higher degrees of anxiety, depression, and sleeplessness compared to the general population. This biased demographic characteristic may limit the generalizability of our findings to the broader population of remote workers. Regarding this limitation, our sample size was relatively small compared with previous studies, although similar sample sizes were sometimes reported in pilot studies (16, 19, 42, 68, 69). To enhance the generalizability of our findings, future studies future studies should aim for larger sample sizes that encompass diverse participants. Finally, although we examined the possible mechanism associated with emodiversity and mental health decline owing to anxiety and depression, these mechanisms are unclear. A comprehensive examination of these possible mechanisms, such as the study of patients with symptoms related to emotion regulation and emotion suppression, should be conducted for utilizing emodiversity-related indicators to detect health deterioration.

## 5. Conclusion

This study aimed to explore the relationship between subjective mental health decline and psycho-physical factors, particularly the diversity of emotional experiences or emodiversity among remote workers. The findings suggest that, even for remote workers in Japan, emodiversity-related indicators can evaluate a decline in mental health such as high anxiety or depressive states, particularly related to emotion dysregulation. Our results may extend the feasibility of emodiversity to remote workers and the Asian region with different cultural backgrounds from Europe and the United States. Future large-scale studies and the establishment of simple methods to monitor emodiversity in everyday life will play an essential role in promoting effective mental health management among remote workers.

## Data availability statement

The datasets presented in this article are not readily available because it is not included in the current ethics approval to submit the dataset. However, the datasets are available from the corresponding author on reasonable request and with additional ethics approval. Requests to access the datasets should be directed to SM, shunsuke.minusa.hd@hitachi.com.

## Ethics statement

The studies involving humans were approved by the internal review board of the Research & Development Group, Hitachi, Ltd. The studies were conducted in accordance with the local legislation and institutional requirements. The participants provided their written informed consent to participate in this study.

## Author contributions

SM conceived, designed, and performed the experiments, analyzed the data and wrote the initial draft of the manuscript. CY and HM supervised the project. All authors interpreted and discussed the experimental results, critically reviewed the manuscript, contributed to the article and approved the submitted version.

## Funding

This work was supported by the Research & Development Group, Hitachi, Ltd. The funder provided support in the form of salaries for authors (SM, CY, and HM) but did not have any additional role in the study design, data collection and analysis, decision to publish, and preparation of the manuscript.

## Conflict of interest

SM, CY, and HM are current employees of the Research & Development Group, Hitachi, Ltd.

## Publisher’s note

All claims expressed in this article are solely those of the authors and do not necessarily represent those of their affiliated organizations, or those of the publisher, the editors and the reviewers. Any product that may be evaluated in this article, or claim that may be made by its manufacturer, is not guaranteed or endorsed by the publisher.

## Footnotes

^1^SF-36® is a registered trademark of Medical Outcomes Trust. (SF-36v2 Standard, Japanese).
