# Supplementary material for: Emodiversity evaluation of remote workers through health monitoring based on intra-day emotion sampling
Source: Front Public Health. 2023 Aug 21;11:1196539. doi: 10.3389/fpubh.2023.1196539 (PMC10475727; doi:10.3389/fpubh.2023.1196539)
Supplement: Supplementary file 1 [file Image_1.pdf]

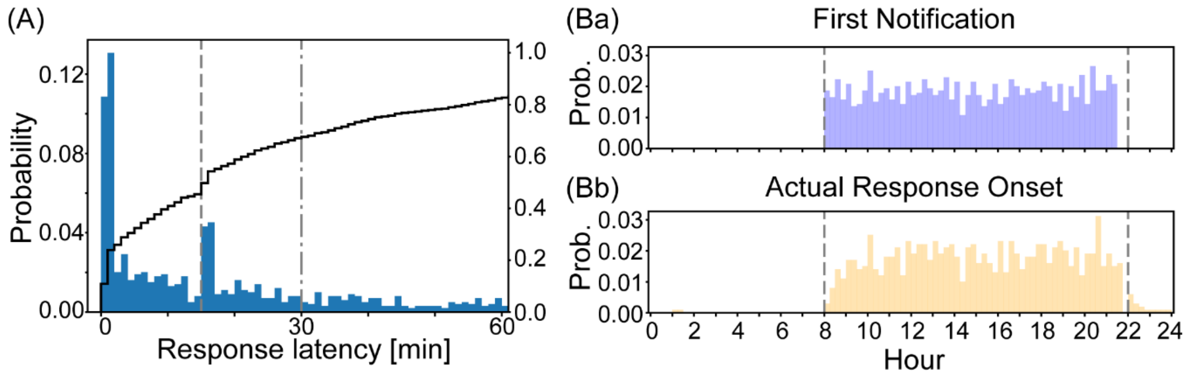

Supplementary Figure 1. Validation of the daily representativeness of Experience Sampling Method (ESM) responses related to notifications. **(A)** Probability (blue histogram) and cumulative (black line) distribution of participants' response latency from ESM notification onset. The first notification onset is aligned with 0 min, and the second reminder is sent 15 min after the onset. Participants are instructed to respond within 30 minutes of notification if possible. Cumulative response rates are 45.5%, 67.1%, and 82.4% at 15, 30, and 60 minutes after notification onset, respectively. This suggests that notifications to the smartphone app are sufficient to trigger user response events. **(Ba)** Probability distributions of ESM notifications (blue) and **(Bb)** participant responses (orange). The notifications and user responses are uniformly distributed, suggesting that the ESM data are sufficiently randomly sampled in a single day.
